# Supplementary material for: Causal Relationship between Aging and Anorexia Nervosa: A White-Matter-Microstructure-Mediated Mendelian Randomization Analysis
Source: Biomedicines. 2024 Aug 16;12(8):1874. doi: 10.3390/biomedicines12081874 (PMC11351342; doi:10.3390/biomedicines12081874)
Supplement: Supplementary file 1 [file biomedicines-12-01874-s001.zip › Supplementary Figure/Figure S4/Singe SNP plot of MR analysis for TL to ubm-b-1469.pdf]

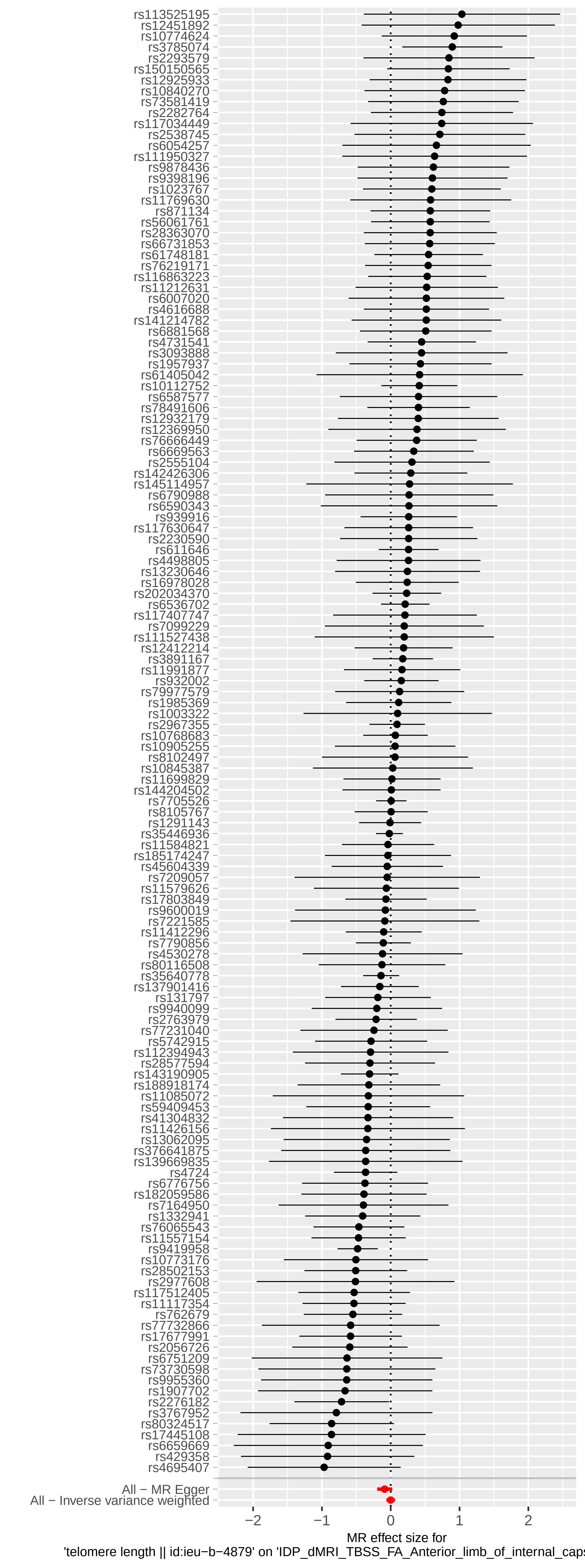

rs113525195
rs12451892
rs10774624
rs3785074
rs2293579
rs150150565
rs12925933
rs10840270
rs73581419
rs2282764
rs117034449
rs2538745
rs6054257
rs111950327
rs9878436
rs9398196
rs1023767
rs11769630
rs871134
rs56061761
rs28363070
rs66731853
rs61748181
rs76219171
rs116863223
rs11212631
rs6007020
rs4616688
rs141214782
rs6881568
rs4731541
rs3093888
rs1957937
rs61405042
rs10112752
rs6587577
rs78491606
rs12932179
rs12369950
rs76666449
rs6669563
rs2555104
rs142426306
rs145114957
rs6790988
rs6590343
rs939916
rs117630647
rs2230590
rs611646
rs4498805
rs13230646
rs16978028
rs202034370
rs6536702
rs117407747
rs7099229
rs111527438
rs12412214
rs3891167
rs11991877
rs932002
rs79977579
rs1985369
rs1003322
rs2967355
rs10768683
rs10905255
rs8102497
rs10845387
rs11699829
rs144204502
rs7705526
rs8105767
rs1291143
rs35446936
rs11584821
rs185174247
rs45604339
rs7209057
rs11579626
rs17803849
rs9600019
rs7221585
rs11412296
rs7790856
rs4530278
rs80116508
rs35640778
rs137901416
rs131797
rs9940099
rs2763979
rs77231040
rs5742915
rs112394943
rs28577594
rs143190905
rs188918174
rs11085072
rs59409453
rs41304832
rs11426156
rs13062095
rs376641875
rs139669835
rs4724
rs6776756
rs182059586
rs7164950
rs1332941
rs76065543
rs11557154
rs9419958
rs10773176
rs28502153
rs2977608
rs117512405
rs11117354
rs762679
rs77732866
rs17677991
rs2056726
rs6751209
rs73730598
rs9955360
rs1907702
rs2276182
rs3767952
rs80324517
rs17445108
rs6659669
rs429358
rs4695407
All − MR Egger
All − Inverse variance weighted
−2
−1
0
1
2
MR effect size for
'telomere length || id:ieu−b−4879' on 'IDP_dMRI_TBSS_FA_Anterior_limb_of_internal_caps
